# Influence of load-bearing wall material properties on building mine-induced dynamic response

**DOI:** 10.1038/s41598-025-15518-3

**Published:** 2025-08-12

**Authors:** Maciej Zajac, Krystyna Kuzniar, Tadeusz Tatara

**Affiliations:** 1 Institute of Technology, University of the National Education Commission, ul. Podchorazych 2, Krakow, 30-084 Poland; 2 Faculty of Civil Engineering, Cracow University of Technology, ul. Warszawska 24, Krakow, 31-155 Poland

**Keywords:** Construction material properties, Building structure, Dynamic response, Mine-induced vibrations, Numerical analysis, Engineering, Civil engineering

## Abstract

Various construction materials are used in contemporary building structures. Modern buildings face impacts like dead, live, snow, and wind load. They may also face extreme conditions, such as seismic activity, which can threaten their safety and functionality. Few publications address how construction material choice affects a building’s dynamic response to seismic impacts, including mining tremors. The main goal of this article is to analyse the effect of load-bearing wall materials on a building’s dynamic response to mine-induced vibrations. Seven materials with different properties were considered: two types of reinforced concrete (standard and high-strength oil palm shell lightweight), cellular concrete, standard brick, and three types of sand-lime bricks. Numerical analysis was based on a low-rise building with a typical wall-bearing structure. The finite element method (FEM) was used to create a three-dimensional (3D) model of the building. The validity of the numerical model was verified through in situ experimental measurements of actual vibrations induced by mining tremors. The numerical predictions showed sufficient accuracy for a building with load-bearing walls made of brick. The study found that the properties of construction materials significantly impact the building’s dynamic behaviour under mine-induced excitations. The natural vibration frequencies of the building varied depending on the wall material used. The maximum vibration acceleration values due to mine tremors also varied significantly with the material type. Differences in maximum acceleration between materials reached up to 56.6% in some tremors. The highest values were observed for cellular concrete walls, which have the lowest stiffness. Using this material in areas affected by mine tremors is not advisable due to the high level of dynamic response, which could cause damage and negatively affect inhabitants. Fourier analyses of the calculated acceleration waveforms revealed differences in dominant vibration frequencies for different materials and tremors with varying energy levels. Although the vibration shapes of buildings with walls made of different materials were similar, the magnitudes of relative displacements differed. The dynamic response is complex, but for weaker tremors, displacement shapes in the longitudinal building direction dominated for all materials. The study concluded that the properties of load-bearing wall materials significantly influence the dynamic behaviour of a building subjected to mine-induced vibrations. This article makes significant scientific contributions and is innovative in several aspects. It presents a validated 3D FEM model for the numerical estimation of a building’s dynamic response to mine-induced kinematic loading. It also compares the effects of various construction materials on the dynamic response of buildings to mine-induced vibrations. By examining seven different materials within a single study, it adds valuable knowledge to the field. Additionally, it provides a practical finding that low-stiffness materials (especially cellular concrete) can lead to increased dynamic responses in mining areas, potentially posing a risk of damage.

## Introduction

Various construction materials are used in contemporary building structures^1–13^. Buildings today face standard impacts like dead, live, snow, and wind load, as well as extreme conditions, including seismic activity, which can be hazardous to their safety and functionality^14–26^.

When selecting materials for new structures or renovating old ones, consider their strength and the pollution generated during production. Recently, there has been a focus on reducing the carbon footprint of construction materials, including greenhouse gas emissions from production, transportation, use, and disposal^27–30^. Addressing this is crucial for sustainable construction and combating climate change. By choosing materials wisely, optimizing production, and encouraging recycling, we can reduce emissions and protect the climate. For example, producing concrete without clinker can significantly reduce CO2 emissions. Limestone calcined clay cement can reduce CO2 emissions by about 40% compared to traditional cement, though it may increase production costs by up to 30%^31,32^.

Seismic activity has been increasing in areas of Poland affected by underground mining, such as the Upper Silesian Coal Basin, the Lublin Coal Basin, and the Legnica-Glogow Copper District. This increase raises the risk to surface infrastructure. Surface buildings vary in construction, age, and condition. Older buildings were constructed using traditional methods^33–36^, while newer buildings are generally made of concrete and reinforced concrete^37,38^. In mining areas, the most common buildings are those with traditional and improved traditional construction, usually brick or small-sized masonry buildings with load-bearing walls. Buildings with concrete and reinforced concrete walls, as well as skeletal structures, have higher resistance to mining shocks. The technical condition of buildings is also considered when assessing their resistance to mining shocks. Historical and heritage buildings in these areas are highly sensitive to vibrations.

An important issue in these areas is determining the behaviours of buildings under shock conditions^39^. Residential buildings must remain within their elastic range because residents do not want to live in buildings with cracked and damaged walls, both structural and partition walls.

High-intensity dynamic loads can damage structural elements that transfer horizontal loads^40^. Common defects include scratches on paint coatings and ceramic wall coverings. Building elements used for spatial planning include non-structural external walls (curtain walls) and non-structural internal walls (partition walls). Other elements enhance the building’s aesthetics and user comfort, such as façade cladding, wall and ceiling plasters and finishes, and paint coatings. This group also includes surface materials like ceramic tiles, carpets, other floor and wall coverings, suspended ceilings, window and door joinery, and design aspects of shear walls and roofing, including the size and placement of openings. Damage can also be caused by devices and technical installations in the building, including lift installations, utility installations, and elements supporting the use of these utilities.

The dynamic response of buildings to kinematic loads involves analysing how structures react to changing forces over time. These forces can be caused by earthquakes or mine-induced vibrations. This analysis is crucial for ensuring the safety and stability of buildings under dynamic conditions. By understanding this response, engineers can design safer and more resilient structures that can withstand the challenges posed by dynamic forces. The behaviour of buildings with load-bearing walls made from different materials significantly influences their dynamic properties. In wind or earthquake-prone zones, load-bearing walls serve as the primary resistance system due to their high in-plane stiffness and strength.

The article focuses on numerically determining and analysing the impact of mining vibrations on a building’s dynamic response, depending on the properties of the load-bearing wall materials. Seven materials with different properties were considered: two types of reinforced concrete (standard and high-strength oil palm shell lightweight), cellular concrete, standard brick, and three types of sand-lime bricks. Numerical analyses were conducted using a low-rise building model with a typical wall-bearing structure and the most intensive surface vibrations. The finite element method (FEM) was used to create a three-dimensional (3D) model of the building. The analysis was conducted within a linear scope, justified by the reactions of residents who do not want their buildings damaged by mining operations, which also affects property value.

The article contributes to science and can be considered innovative in the following aspects:

1. It presents a validated 3D FEM model for the numerical estimation of a building’s dynamic response to mine-induced kinematic loading.
2. It compares the effects of various building construction materials on mine-induced building dynamic response. The comparison of seven different materials within a single study contributes to the existing knowledge in this field.
3. It quantitatively demonstrates the influence of load-bearing wall materials on the building’s natural frequencies, acceleration responses, and dominant response frequencies.
4. It provides a practical finding that low-stiffness materials (especially cellular concrete) can lead to increased dynamic response in mining areas, potentially posing a damage risk.

This study provides important data that should be considered in structural design and material selection, particularly in areas with intensive mining activities.

## Materials and methods

### Analysed building construction materials

To analyse the influence of load-bearing wall materials on a building’s response to mine-induced excitations, typical materials were selected^39^. These materials are standard reinforced concrete (SRC), high-strength oil palm shell lightweight reinforced concrete (LRC), cellular concrete (CC), standard brick (B), and three types of sand-lime bricks (SLB1, SLB2, and SLB3).

These materials have very different properties, such as elastic modulus, density, and Poisson’s ratio. The characteristics of the seven load-bearing wall materials used in the dynamic analyses are summarized in Table 1. The table also includes the thicknesses of the wall load-bearing layers for each material.

**Table 1:** The most important parameters (properties) of analysed load-bearing wall materials.

	Material						
	SRC	LRC	CC	B	SLB1	SLB2	SLB3
Elastic modulus [GPa]	31.0	13.4	1.80	2.85	5.56	6.68	6.94
Poisson’s Ratio [-]	0.25	0.20	0.25	0.25	0.23	0.21	0.23
Density [kg/m^3^]	2500	1900	600	1800	1820	1810	1730
Load-bearing wall thickness [m]	0.15	0.15	0.25	0.25	0.18	0.18	0.24

All external and internal load-bearing walls have two layers of cement-lime plaster. The thicknesses for external walls are 0.015 m and 0.02 m. The thicknesses for internal walls are 0.015 m each. Additionally, each external load-bearing wall has a thermal insulating layer made of styrofoam, with a thickness of 0.15 m.

The remaining parts of the building are composed of concrete (strip footing), reinforced concrete (ceilings, ceiling ties, foundation tie rods, foundation walls, lintels, stairs, prefabricated roof panels), and brick (partition walls, supporting knee walls).

### Numerical analysis using 3D FEM building model

In this article, a two-story office building without a basement, located in the mining-induced seismic region of the Upper Silesian Coal Basin in Poland, was used for analysis. The study focused on the influence of load-bearing wall material properties on the building’s dynamic response to mine-induced vibrations. The building’s dimensions are 12.7 m in the transverse (x) direction, 29.9 m in the longitudinal (y) direction, and approximately 7.3 m in height.

The focus was on low-rise buildings as representatives of this class of structures. Low-rise buildings constitute a significant part of the built environment in areas affected by anthropogenic seismic activity. This includes both single-family residential buildings and structures that are part of the surface infrastructure of mines. Administrative buildings in mining areas often exhibit similar construction characteristics to those analysed in this article. This formed the basis for analysing this type of construction. Future research plans aim to expand the scope to include taller typical buildings with 5 and 12 stories erected in towns within mining regions.

**Fig. 1:**
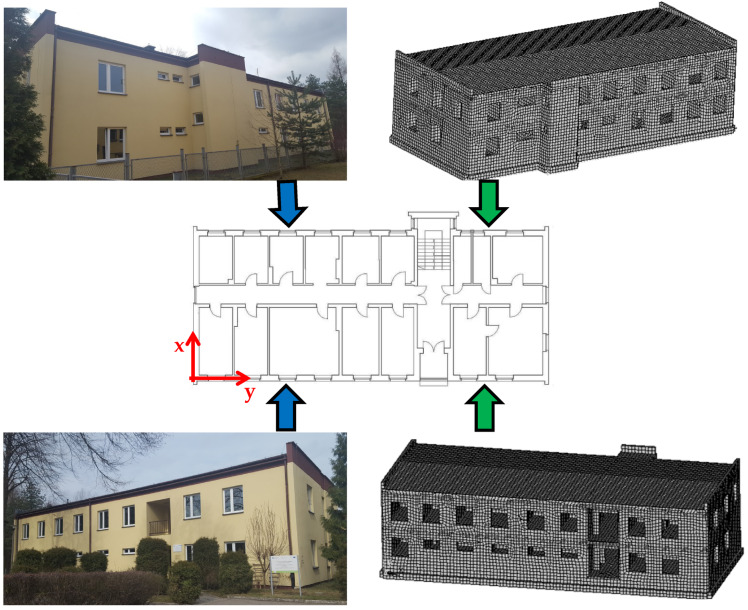
Building second floor plan together with two-side facade views of the actual analysed building and 3D FEM model.

The structure was numerically modelled using ANSYS 2022 R1 Mechanical software^41^. Numerical analyses were based on detailed architectural documentation, including floor plans of both ground and second floors, and cross-sections of foundations and walls. The second-floor plan, two-side facade views, and the 3D FEM model of the building are shown in Fig. 1.

The building was supported on strip foundations. The foundation footings were modelled as reinforced concrete beams with cross-sections of 0.4 m × 0.7 m and 0.4 m × 0.5 m. The foundation tie beams had square cross-sections of 0.3 m × 0.3 m to stiffen the foundation due to mining-induced vibrations.

All main structural elements were modelled using two types of finite elements: 4-node elastic shell elements (SHELL181) with six degrees of freedom per node, and 2-node beam elements (BEAM188) with six degrees of freedom. Shell elements were used for the roof, ceilings, load-bearing walls, and partition walls. Beam elements were used for linear structural members, specifically the foundation footings, foundation tie beams, and ceiling ties. The slabs between floors and the roof were also represented by shell elements, with thicknesses reflecting actual structural details.

Linear-elastic behaviour of structural materials was assumed, justified by previous in-situ inspections showing no structural damage indicative of nonlinear material response. The building with brick load-bearing walls was studied over several years during which it was repeatedly subjected to dynamic loads of varying mining tremors intensity. Observations of the building’s technical condition did not reveal any structural damage such as cracks in load-bearing walls, lintels, floors, or foundations. Only so-called architectural damage was observed, in the form of minor cracks in plaster and paint coatings. Elastic load-bearing material behaviour and building dynamic responses in the elastic range have been found in the case of the actual building. That was the reason why non-linear analyses were not conducted.

The influence of the soil substrate was considered by adopting soil type class B according to Eurocode 8^42^, based on geotechnical data. From in situ geotechnical measurements (strength of the soil, its type, wave velocity), soil category B was adopted, as the subsoil in the analysed area consists of medium dense sands. The analysis of soil borehole data at the actual building site indicates that the shear wave velocity is 200 m/s. These geotechnical parameters are consistent with the B category. The soil conditions are typical and representative for the Upper Silesian Coal Basin, as well as for other mining regions, such as the Legnica-Glogow Copper District. Soil-structure interaction was included using COMBIN14 spring-damper elements. Their stiffnesses were calculated based on the layered soil model proposed by Savinov^43^. A dynamic soil reaction parameter (Cz) of 50 MPa was assumed. Additionally, soil damping properties were considered in calculations, using typical values for medium-dense sand (soil density 1800 kg/m³, shear wave velocity 200 m/s).

Structural responses to dynamic excitations in horizontal directions (x, y) were determined numerically using ANSYS software. The Newmark integration scheme and Rayleigh damping model with a 5% damping ratio were applied.

The validity of the numerical model was verified based on experimental measurements of actual vibrations induced by mining tremors in the Upper Silesian Coal Basin in Poland^44^. Records of horizontal vibration acceleration components in the x and y directions were used. These were registered simultaneously in situ on the ground next to the building and inside the building at the ground floor level. Using the proposed model and the measured ground vibrations as excitation, the building vibrations were calculated at the sensor location. Sufficient accuracy of numerical predictions for a building with load-bearing walls made of brick was confirmed^39,44^.

## Results and discussion

The influence of load-bearing wall material properties on a building’s dynamic response to mine-induced vibrations was analysed. Data from in situ measured free-field vibrations near the building were used as excitation. The focus was on horizontal vibrations in the x (transverse) and y (longitudinal) directions (see Fig. 1).

The building model’s kinematic load includes horizontal x and y vibration components from three mining tremors recorded in the Upper Silesian Coal Basin. These tremors, labelled RB1, RB2, and RB3, have different energy levels and epicentre distances. The energy ranges from 8 × 10^5^ J to 8 × 10^7^ J, and the epicentre distances range from 855 m to 1408 m. The characteristics of the rock bursts are listed in Table 2.

**Table 2:** Parameters of the discussed rock bursts.

Rock burst parameters	Rock burst		
	RB1	RB2	RB3
Energy [J]	8 × 10^7^	3 × 10^6^	8 × 10^5^
Epicentral distance [m]	899	1408	855

**Fig. 2:**
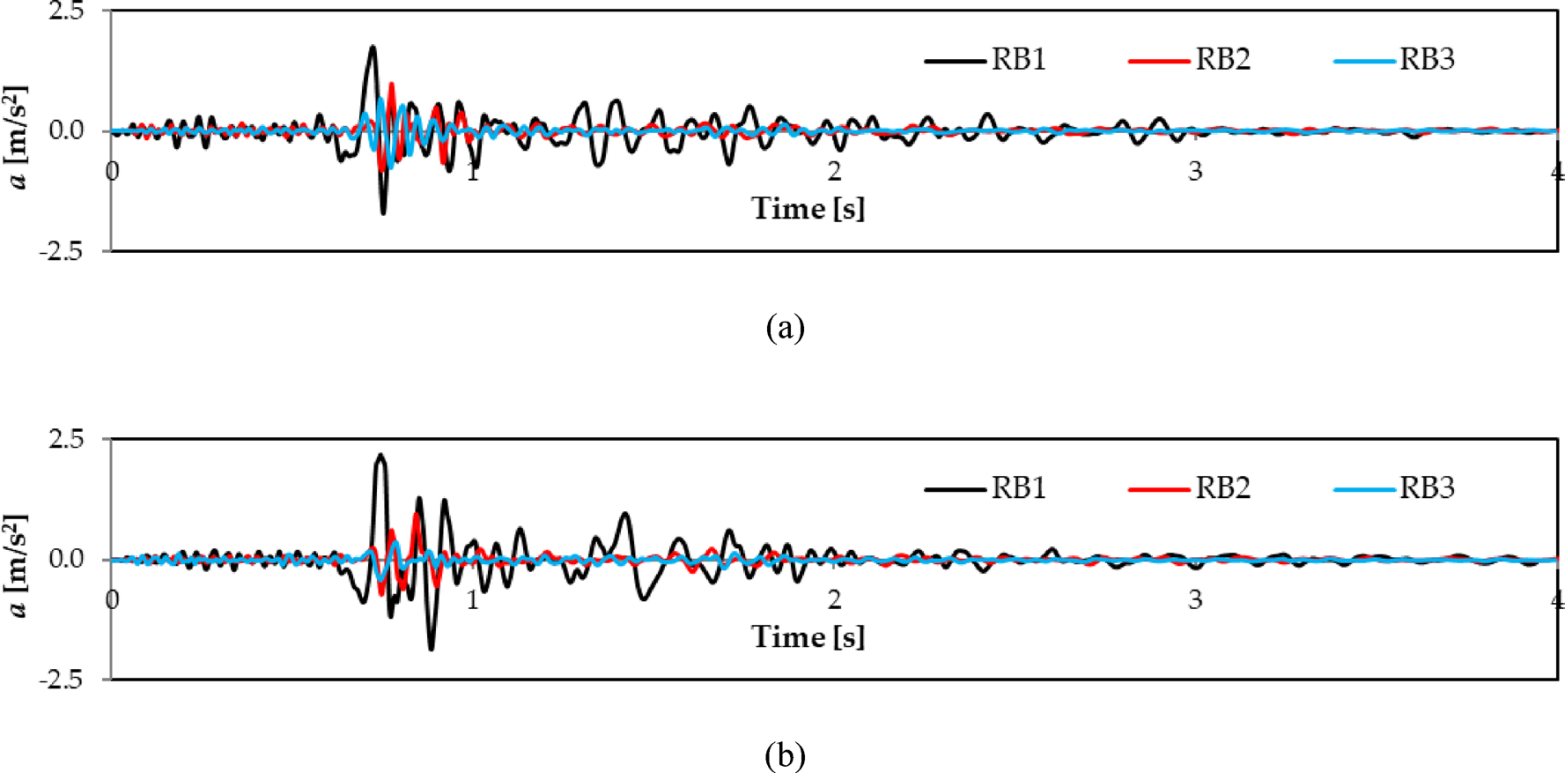
In situ measured free-field time history vibration induced by the three discussed rock bursts: (**a**) horizontal transverse direction (x); (**b**) horizontal longitudinal direction (y).

**Fig. 3:**
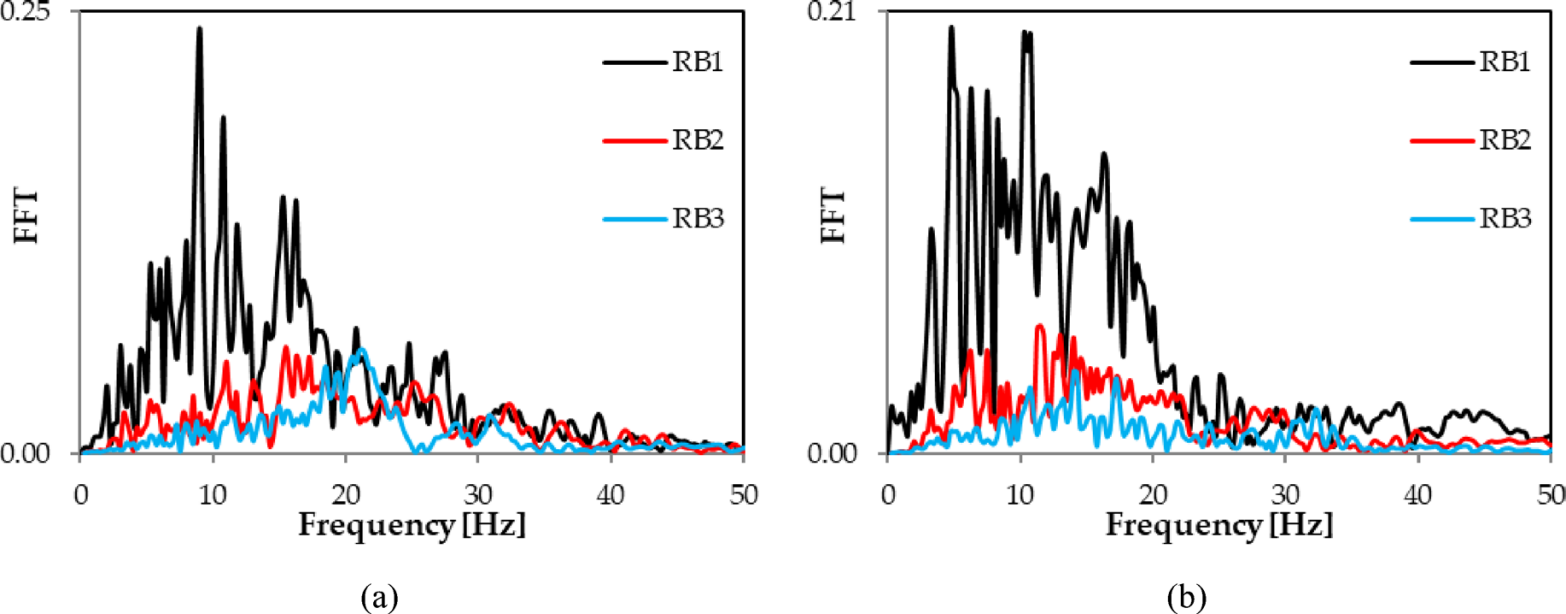
Fourier spectra (FFT) corresponding to free-field time history vibration induced by the three discussed rock bursts: (**a**) horizontal transverse direction (x); (**b**) horizontal longitudinal direction (y).

The recorded waveforms of the horizontal x and y acceleration components for the RB1, RB2, and RB3 tremors are shown in Fig. 2a and b. The corresponding Fourier spectra (FFT) based on these recorded vibrations are displayed in Fig. 3.

The recorded horizontal vibration components in the x direction show significant differences in maximum acceleration values and dominant frequencies. The maximum acceleration for the RB1 shock, a high-energy tremor, reaches 1.76 m/s². In contrast, the values for RB2 and RB3 are lower at 0.97 m/s² and 0.78 m/s², respectively. In the y direction, the RB1 tremor exhibits a higher maximum acceleration of approximately 2.2 m/s². For RB2 and RB3, the y component values are 0.95 m/s² and 0.42 m/s², respectively. RB2 and RB3 are classified as low-energy shocks (see Fig. 2).

FFT analyses of the horizontal x and y vibration components have identified dominant frequency bands. For the high-energy RB1 shock, the x component has two bands: 8.5–12 Hz and 15–16 Hz. Low-energy shocks RB2 and RB3 display x component frequencies that range from 12 to 25 Hz. The y component for the RB1 shock spans a broad frequency range of 3–20 Hz. For RB2, it ranges from 5 to 15 Hz, whereas for RB3, it shifts to 12–17 Hz (see Fig. 3).

**Fig. 4:**
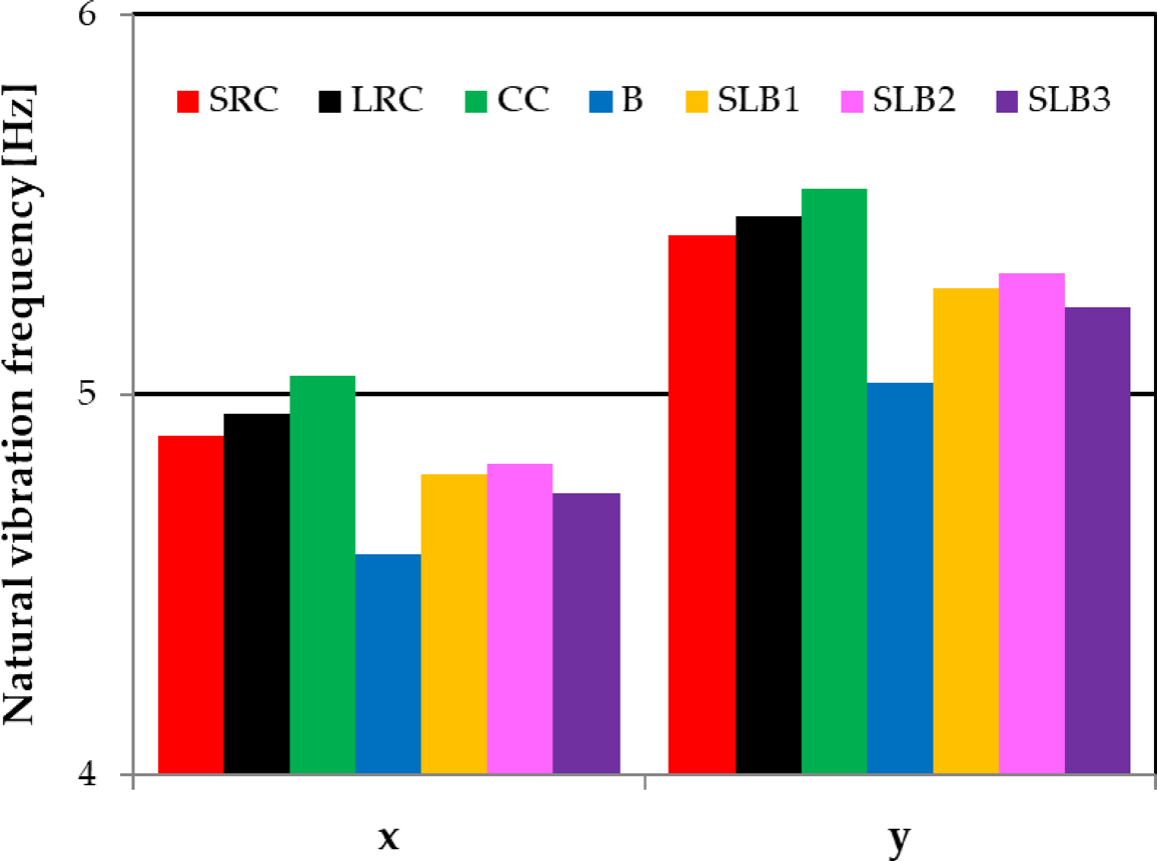
Building natural horizontal vibration frequencies (directions x and y) depending on the material variant of building load-bearing walls.

The building’s dynamic response to three kinematic excitations was analysed using seven load-bearing wall materials (see Table 1). Initially, the building’s natural frequencies in the x and y directions were determined^39^. The calculated natural frequencies for buildings with different wall materials are shown in Fig. 4.

In the x direction, the highest natural frequency is for cellular concrete walls (5.05 Hz) and the lowest for masonry walls (4.58 Hz). For other materials, the values are below 5 Hz (see Fig. 4). In the y direction, buildings with all wall materials have natural frequencies above 5 Hz, indicating greater stiffness. The highest value is for cellular concrete walls (5.54 Hz) and the lowest for masonry walls (5.03 Hz). Furthermore, for other materials, the y direction frequencies are approximately 10% higher than those in the x direction (see Fig. 4). It is essential to consider the differences in stiffness between directions and assumed load-bearing wall materials when conducting dynamic analysis and structural design.

**Fig. 5:**
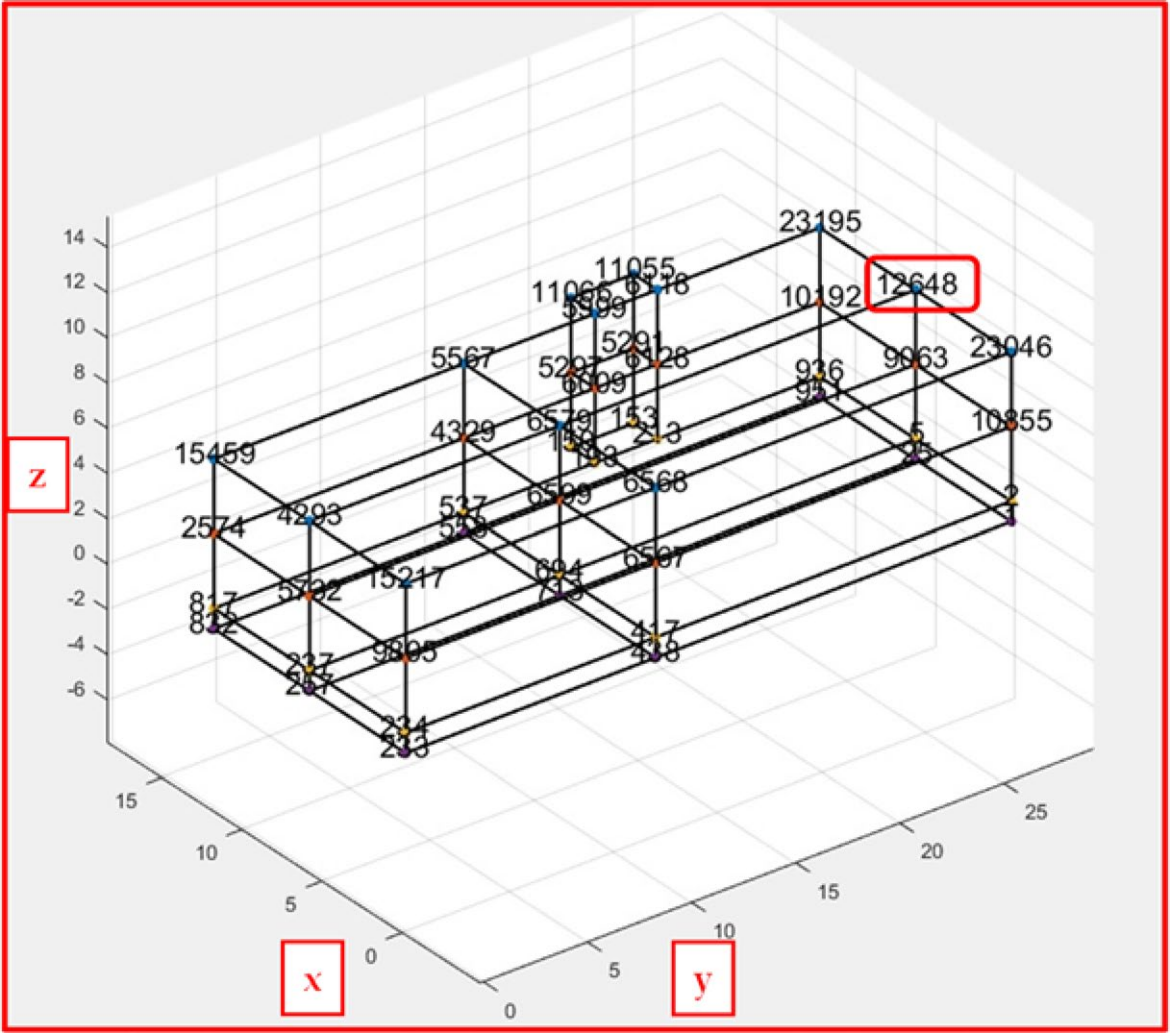
Illustration of the selected node location.

The dynamic response for the wall materials was calculated at selected points (see Fig. 5) for three mining tremors.

**Fig. 6:**
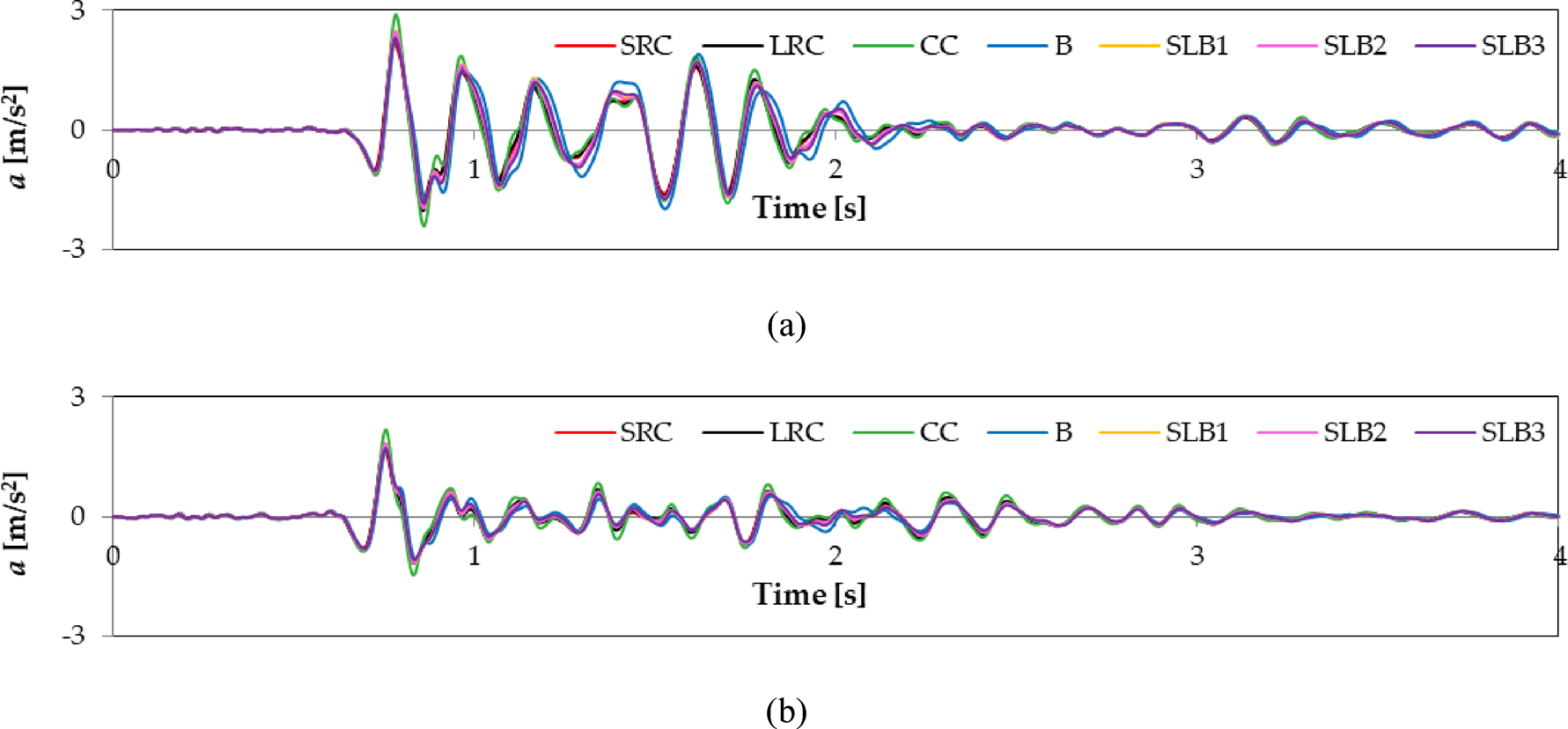
RB1 mine-induced building dynamic response depending on load-bearing wall material properties: (**a**) direction x; (**b**) direction y.

**Fig. 7:**
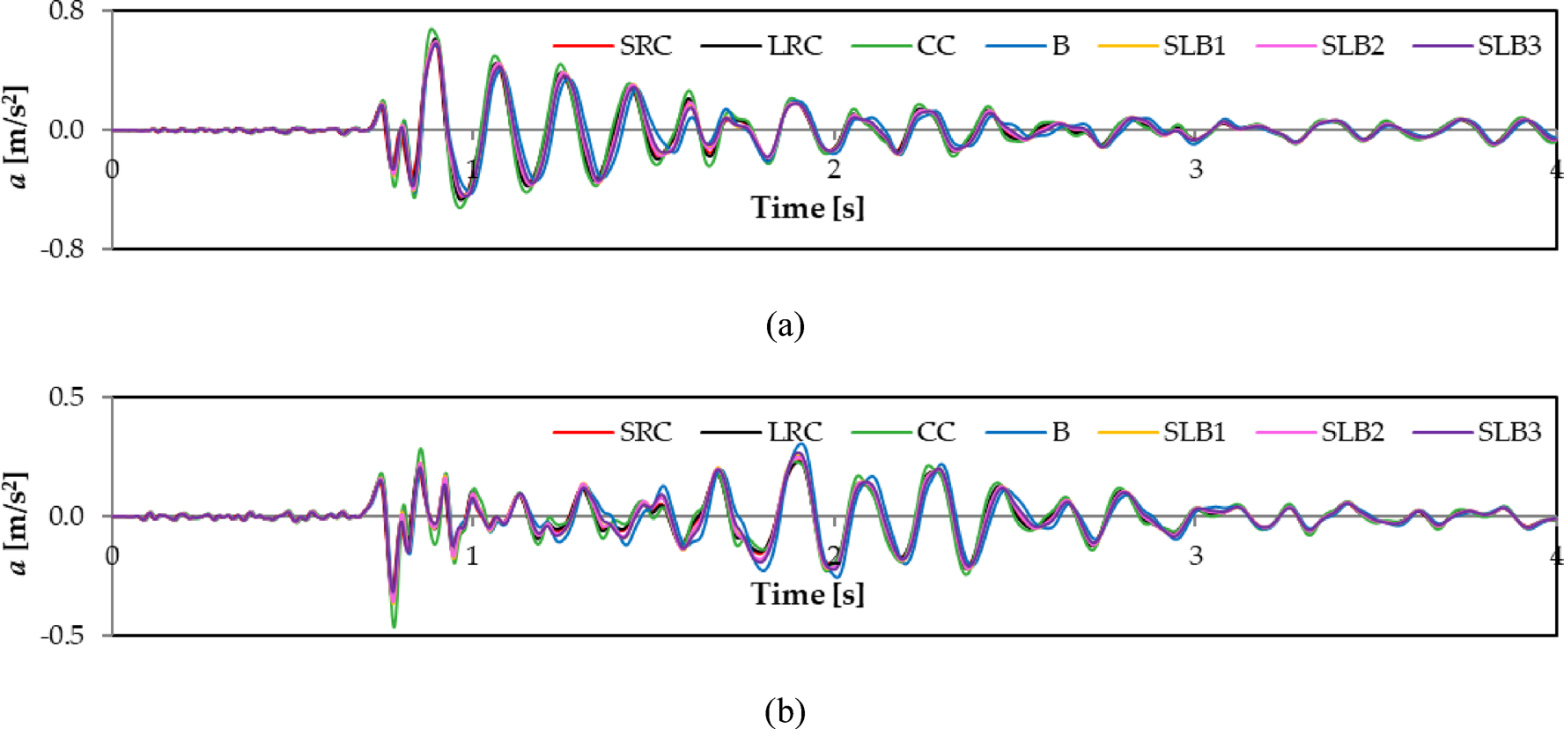
RB2 mine-induced building dynamic response depending on load-bearing wall material properties: (**a**) direction x; (**b**) direction y.

**Fig. 8:**
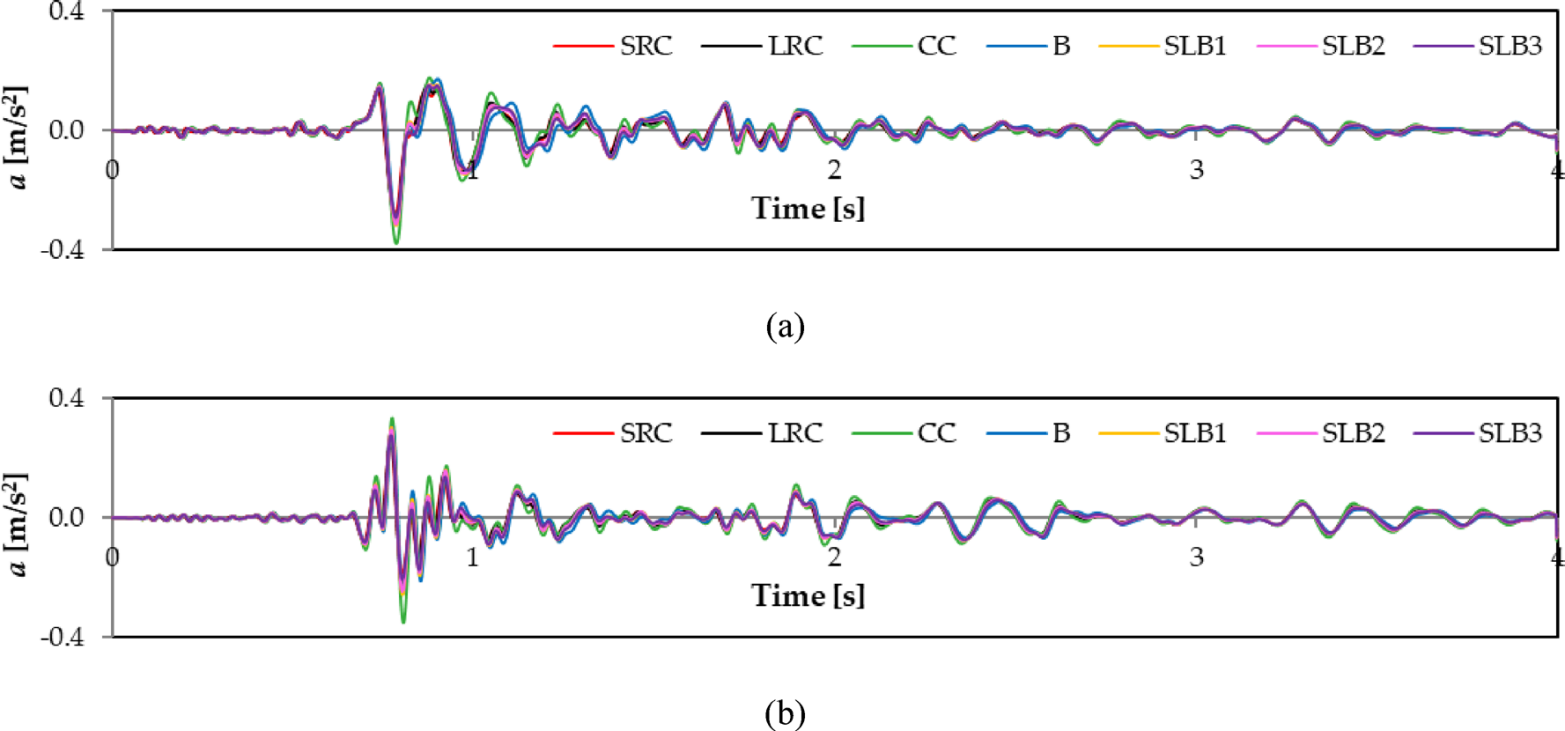
RB3 mine-induced building dynamic response depending on load-bearing wall material properties: (**a**) direction x; (**b**) direction y.

**Fig. 9:**
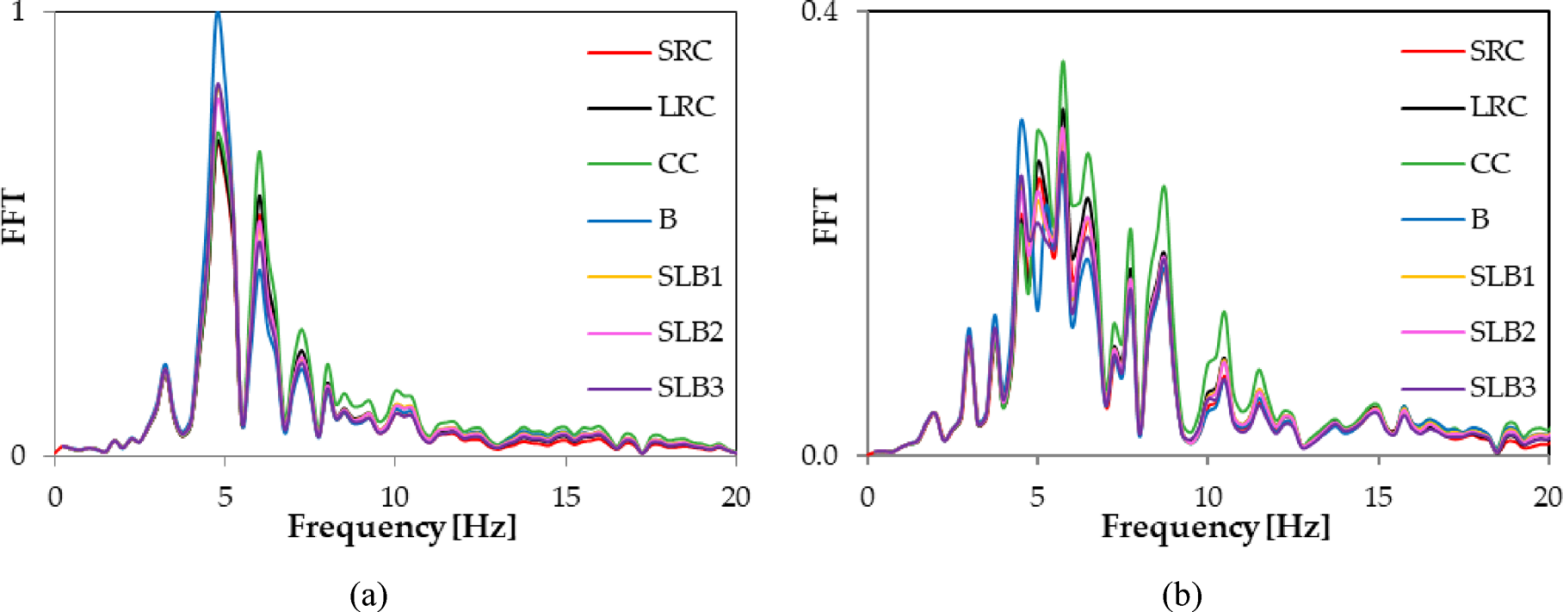
Comparison of Fourier spectra (FFT) corresponding to RB1 mine-induced building dynamic response for various structural materials: (**a**) direction x; (**b**) direction y.

**Fig. 10:**
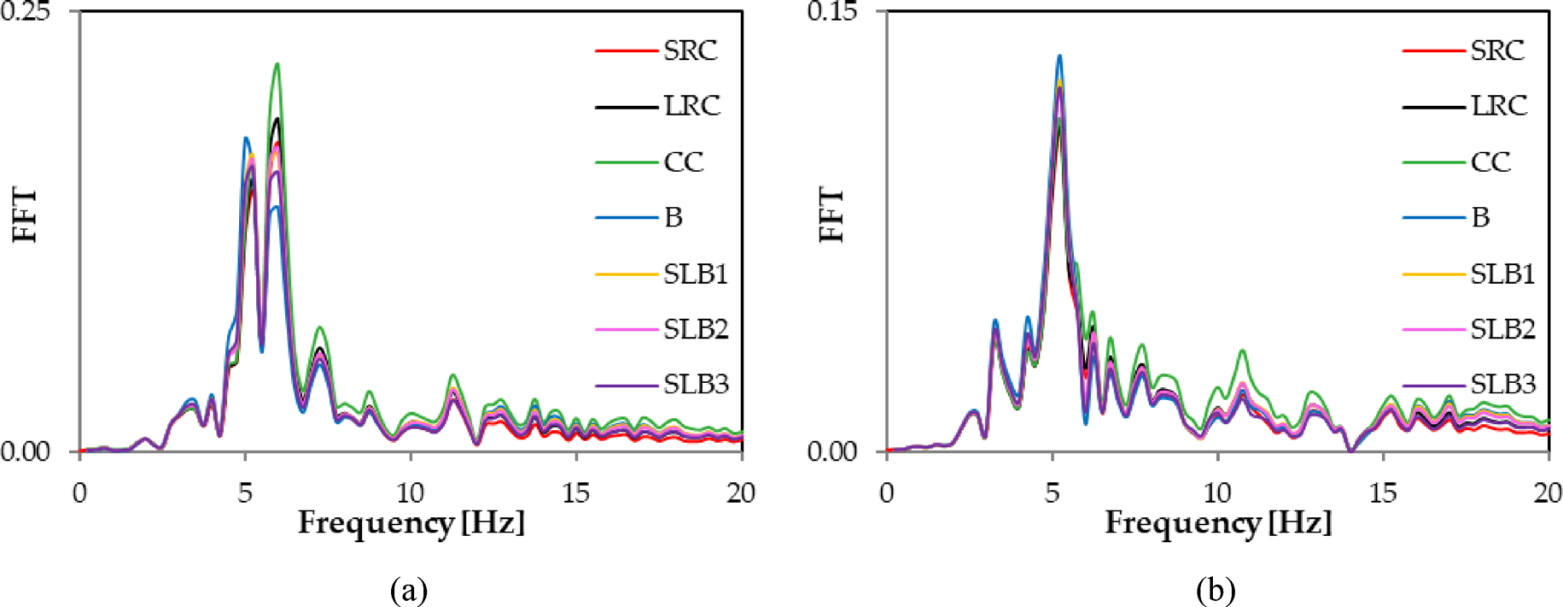
Comparison of Fourier spectra (FFT) corresponding to RB2 mine-induced building dynamic response for various structural materials: (**a**) direction x; (**b**) direction y.

**Fig. 11:**
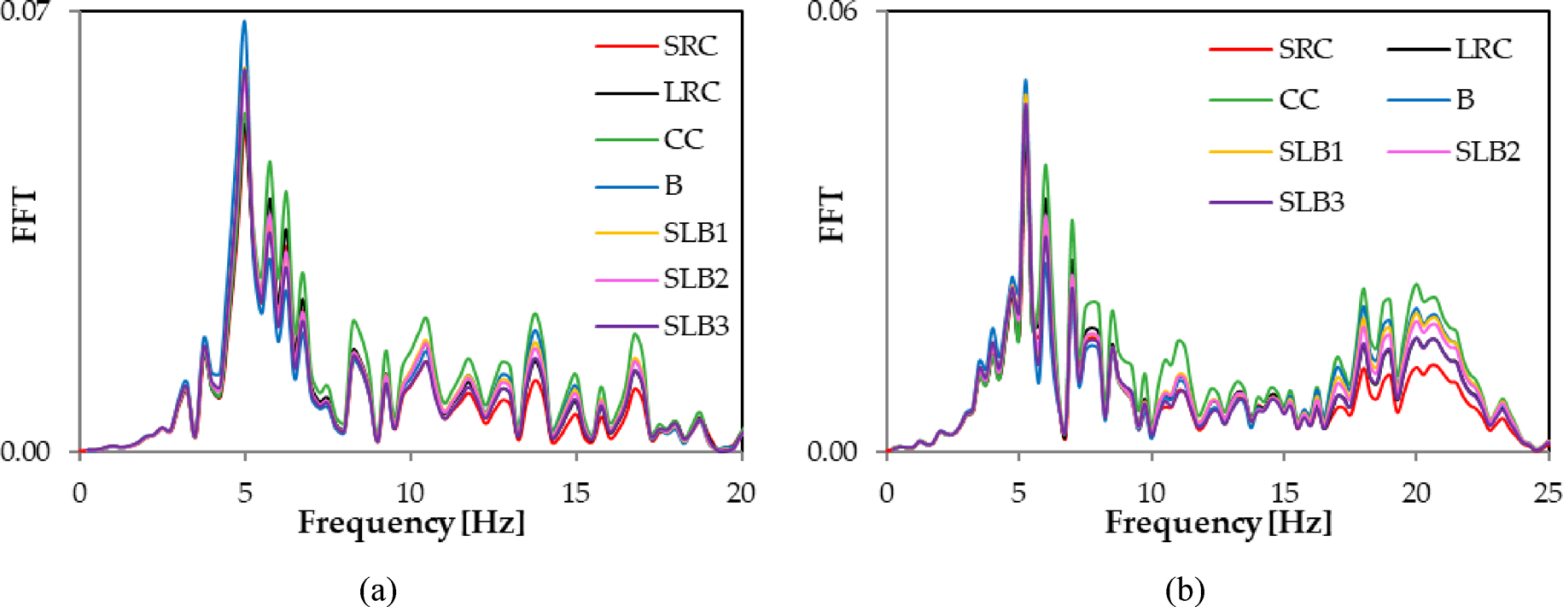
Comparison of Fourier spectra (FFT) corresponding to RB3 mine-induced building dynamic response for various structural materials: (**a**) direction x; (**b**) direction y.

For example, Figs. 6 and 7, and 8 show the calculated vibration acceleration waveforms in the horizontal directions x and y at the selected node No. 12,648 (see Fig. 5), located at the top of the external load-bearing wall. The corresponding Fourier spectra (FFT) are presented in Figs. 9, 10, and 11. The peak acceleration and dominant vibration frequency values for node No. 12,648, depending on the wall materials and the three rock bursts, are summarised in Table 3.

**Table 3:** Values of peak acceleration and dominant frequency of vibration determined for structure dynamic responses depending on the analysed wall-bearing wall materials.

	Rock burst	Direction	Material						
			SRC	LRC	CC	B	SLB1	SLB2	SLB3
Peak acceleration value [m/s^2^]	RB1	x	2.222	2.441	2.904	2.398	2.488	2.464	2.314
		y	1.679	1.835	2.187	1.715	1.829	1.819	1.714
	RB2	x	0.596	0.614	0.679	0.565	0.591	0.595	0.582
		y	0.295	0.347	0.462	0.344	0.364	0.356	0.315
	RB3	x	0.278	0.306	0.377	0.307	0.317	0.312	0.289
		y	0.247	0.279	0.349	0.305	0.303	0.295	0.276
Dominant frequency value [Hz]	RB1	x	4.75	4.75	4.75	4.75	4.75	4.75	4.75
		y	5.75	5.75	5.75	4.50	5.75	5.75	5.75
	RB2	x	6.00	6.00	6.00	5.00	6.00	6.00	5.25
		y	5.25	5.25	5.25	5.25	5.25	5.25	5.25
	RB3	x	5.00	5.00	5.00	5.00	5.00	5.00	5.00
		y	5.25	5.25	5.25	5.25	5.25	5.25	5.25

For node No. 12,648, the highest vibration acceleration in the x direction was observed for cellular concrete walls due to their low stiffness. Walls with lower stiffness (such as cellural concrete) dampen vibrations in this transverse x direction less effectively, resulting in higher acceleration values ​​and potentially increasing the risk of damage or user discomfort.

The vibration acceleration values in the y direction are smaller than those in the x direction owing to the building’s greater stiffness and dynamic resistance in the y direction. For all mining shocks, the building’s dynamic responses are greater in the x direction for all wall materials (see Figs. 6, 7 and 8; Table 3).

The impact of wall material on maximum response values was assessed. Comparing acceleration values at node No. 12,648 for different wall materials shows the greatest differences for RB2 and RB3 shocks. For RB1, the maximum differences in the x and y directions are 31% and 30%, respectively. The greatest differences in the y direction for RB2 and RB3 are 56.6% and 41.3%, respectively. The smallest difference in the x direction for RB2 is 20%.

FFT analyses of the x and y acceleration components at node No. 12,648 showed differences in dominant frequencies. For RB1, the y direction frequency increased from 4.5 Hz (traditional masonry brick wall) to 5.75 Hz for other wall materials. For RB2, the x direction frequency changed, with the smallest values being 5 Hz (traditional masonry brick wall) and 5.25 Hz (sand-lime brick wall SLB3). For other materials, the dominant frequency was 6 Hz. For RB3, the dominant frequency values in the x direction were the same, and a similar pattern was seen in the y direction (see Table 3; Figs. 9 and 10, and 11). Spectral analysis (FFT) of the accelerations revealed that changing the wall material affects not only the extreme values ​​of the response, but also the dominant vibration frequencies. Shifts in these frequencies can impact how the building responds to specific types of rock bursts, including the occurrence of resonance phenomena.

**Fig. 12:**
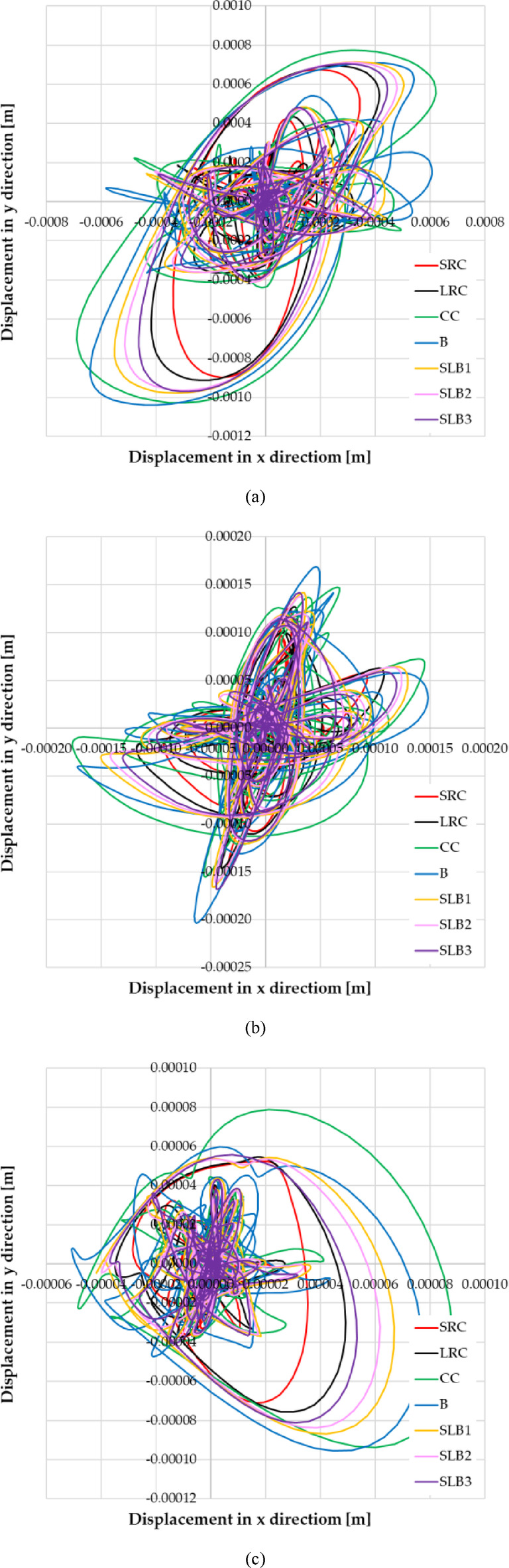
Trajectories of the end of the resultant relative vibration displacement vector at node 12,648 depending on applied structural wall-bearing wall material in the case of the rock burst: (**a**) RB1; (**b**) RB2; (**c**) RB3.

Furthermore, the trajectories of the end of the resultant relative vibration displacement vector at node No. 12,648 were calculated for different wall materials for RB1, RB2, and RB3 (see Fig. 12). Comparing these trajectories shows the complexity of the dynamic response, with no clear dominant vibration direction. There are significant differences in displacements for buildings with different wall materials.

**Fig. 13:**
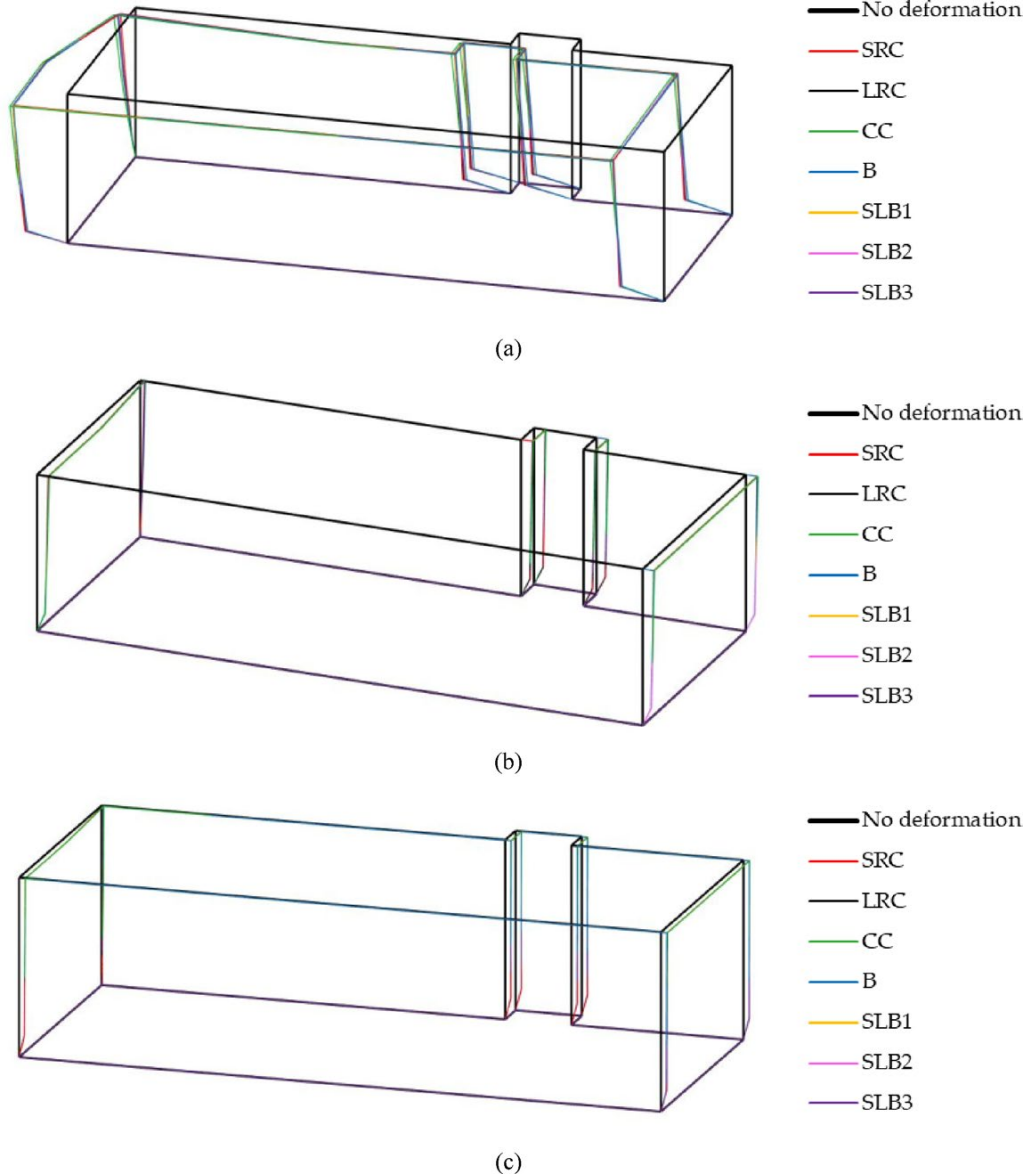
Three-dimensional form of building displacement dynamic response depending on applied structural wall-bearing wall material in the case of: (**a**) the rock burst RB1, at time t = 0.77 s; (**b**) the rock burst RB2, at time t = 0.98 s; (**c**) the rock burst RB3, at time t = 0.78 s.

The final numerical analysis involved calculating and comparing the three-dimensional displacement response of the building for different wall materials during the RB1, RB2, and RB3 tremors. These vibration forms at selected moments are shown in Fig. 13. Displacement values were scaled 1000 times for better visualization. The vibration forms were superimposed on the undeformed model (see Fig. 13). The analysis shows that for the RB1 tremor, the vibration form is complex. For RB2 and RB3, vibrations in the y direction dominate. While the vibration forms for different wall materials are similar, the displacement sizes vary.

## Conclusions

The article presents results from numerical dynamic analyses using a 3D FEM model of a building. Seven load-bearing wall materials were evaluated to compare the building’s dynamic behaviour. The study focuses on the impact of mining ground-borne vibrations on the building’s dynamic response, based on wall material properties. The model was subjected to three excitations from recorded horizontal ground vibration accelerations. These shocks had varying energy levels, maximum acceleration values, and epicentre distances.

The research on the impact of different wall materials on the dynamic response of low-rise buildings leads to the following key conclusions:

1. Natural Vibration Frequencies: The building’s natural vibration frequencies vary with wall material. It’s stiffer in the y direction than in the x direction, causing different frequency values. Material differences led to about a 10% variation in frequencies.
2. Maximum Vibration Acceleration of Free-Field Horizontal Components: For high-energy tremors, the maximum ground vibration accelerations reach 1.76 m/s² (x) and 2.2 m/s² (y). For low-energy tremors, these values are lower, below 1 m/s².
3. Dominant Frequencies of Free-Field Vibration Components: FFT analyses identified dominant frequency bands in the x and y components. For high-energy tremors, the x component has frequency bands at 8.5–12 Hz and 15–16 Hz. For low-energy tremors, it ranges from 12 Hz to 25 Hz. The y component ranges from 3 Hz to 20 Hz for high-energy and from 5 Hz to 17 Hz for low-energy tremors.
4. Maximum Vibration Acceleration Response of the Building: Maximum vibration acceleration due to mine tremors varies with material type. Cellular concrete (CC) walls, being the least stiff, showed the highest values. Consequently, using CC in tremor-prone areas is not recommended due to high dynamic response, which may cause damage. The differences in maximum acceleration between materials reach as high as 56.6%.
5. Dominant Response Vibration Frequencies: Fourier analyses showed differences in dominant vibration frequencies for various materials and tremor energy levels. For high-energy tremors, the greatest difference in dominant frequency occurs between building with cellular concrete walls and building with masonry walls in the y direction, amounting to 28%.
6. Displacement Mode Shapes: Vibration shapes of buildings with different wall materials were similar, but displacement magnitudes varied. The dynamic response is complex, with y direction displacements dominating for weaker tremors. Numerical analyses indicate that for weaker shocks, transverse y direction displacements dominate for all wall materials.

In conclusion, the study found that wall material properties significantly influence a building’s dynamic behaviour under mine-induced vibrations. Therefore, selecting the appropriate wall material can contribute to improving the safety of the structure and the comfort of its users under conditions of dynamic impact.

## Data Availability

Data can be provided from the corresponding author upon a reasonable request.
